# Gulf war illness-related chemicals increase CD11b/c^+^ monocyte infiltration into the liver and aggravate hepatic cholestasis in a rodent model

**DOI:** 10.1038/s41598-018-31599-9

**Published:** 2018-09-03

**Authors:** Anca D. Petrescu, Stephanie Grant, Gabriel Frampton, Matthew McMillin, Jessica Kain, Maheedhar Kodali, Ashok K. Shetty, Sharon DeMorrow

**Affiliations:** 1 Department of Medical Physiology, Texas A&M College of Medicine, Temple, 76504 USA; 2 Central Texas Veterans Healthcare System, Temple, 76504 USA; 3 Institute for Regenerative Medicine, Department of Molecular and Cellular Medicine, Texas A&M College of Medicine, College Station, 77843 USA

## Abstract

Gulf War Illness (GWI) is a chronic multisymptom disorder affecting veterans of the 1990–91 Gulf war. GWI was linked with exposure to chemicals including the nerve gas prophylactic drug pyridostigmine-bromide (PB) and pesticides (DEET, permethrin). Veterans with GWI exhibit prolonged, low-level systemic inflammation, though whether this impacts the liver is unknown. While no evidence exists that GWI-related chemicals are hepatotoxic, the prolonged inflammation may alter the liver’s response to insults such as cholestatic injury. We assessed the effects of GWI-related chemicals on macrophage infiltration and its subsequent influence on hepatic cholestasis. Sprague Dawley rats were treated daily with PB, DEET and permethrin followed by 15 minutes of restraint stress for 28 days. Ten weeks afterward, GWI rats or naïve age-matched controls underwent bile duct ligation (BDL) or sham surgeries. Exposure to GWI-related chemicals alone increased IL-6, and CD11b^+^F4/80^−^ macrophages in the liver, with no effect on biliary mass or hepatic fibrosis. However, pre-exposure to GWI-related chemicals enhanced biliary hyperplasia and fibrogenesis caused by BDL, compared to naïve rats undergoing the same surgery. These data suggest that GWI patients could be predisposed to developing worse liver pathology due to sustained low-level inflammation of the liver when compared to patients without GWI.

## Introduction

Gulf War Illness (GWI) is a medical condition affecting the veterans of the 1990–1991 Persian Gulf war, and includes a cluster of chronic symptoms such as headaches, joint pain, muscle pain, indigestion, fatigue, insomnia, dizziness, respiratory disorders, cognitive and memory problems^1^. In 2004, the Research Advisory Committee on Gulf War Veterans’ Illnesses suggested that this cluster of symptoms could not be explained by wartime stress or psychiatric illness alone, but are linked to exposure to neurotoxins, such as the nerve gas prophylactic drug pyridostigmine bromide (PB), and pesticides, especially DEET and permethrin, or more likely a combination of these chemicals^2^.

Aside from the obvious and well-known medical symptoms typical for GWI, a number of new observations and discoveries have been made by research on experimental models of this syndrome, especially those using rodents, rats in particular, subjected to GWI-related chemicals^3–10^. It was demonstrated that GWI-related chemicals can cause chronic oxidative stress, mitochondrial dysfunction and ultimately inflammation of certain areas of the brain, in association with elevated oxidative stress and inflammation at the systemic level^9^. Aspects of mood change and memory impairment due to exposure to GWI-related chemicals were also demonstrated in animal models of GWI^7,10^. Several immune anomalies have been linked to GWI^11–13^. For example, when GWI veterans and healthy volunteers were compared, the percentage of B cells was significantly elevated in patients versus controls and a high number of patients had more T-cells than normal^12^. Natural killer cell activity was found to be significantly reduced in GWI patients as compared to healthy controls^12,13^. While no autoimmune diseases or particular allergies have been identified in patients with GWI, recent research has suggested elevated levels of the inflammatory markers IL-2, IFN-γ, CCL11, FGF, IL-5, IL-17, IL-33, Leptin, C-reactive protein, and MMP-9 in the plasma of GWI patients^11,14–16^.

While liver damage has not been reported with GWI-related chemicals to-date, many liver diseases share a longstanding relationship with inflammation and therefore may be influenced by pre-exposure to GWI-related chemicals, in particular those liver diseases that result in the development of liver fibrosis. Hepatic fibrogenesis is usually preceded by chronic inflammation and leads to an excessive buildup of extracellular matrix proteins in most types of chronic liver diseases^17,18^. Many cell types in the liver contribute to the deposition of these extracellular matrix proteins, including proliferating cholangiocytes and activated hepatic stellate cells (HSCs). Therefore, it is conceivable that chronic exposure to GWI-related chemicals may alter the response of either of these cell types, exacerbating the resulting liver fibrosis. Further, in a subset of chronic liver diseases termed cholangiopathies, cholangiocytes proliferate and become reactive, secreting chemokines that attract and recruit pro-inflammatory monocyte/macrophages causing hepatic inflammation^19^. Concurrently, many quiescent HSCs are activated, becoming more proliferative and undergoing phenotypical and morphological changes^20^. The activated HSCs start producing and secreting extracellular matrix proteins causing hepatic fibrosis and functional dysregulation of hepatocytes. Removal of the injurious agents results in the clearance of activated HSCs by the cytotoxic actions of natural killer cells and regression of fibrosis. Those activated HSCs that escape removal by natural killer cells may then revert to a quiescent-like phenotype and downregulate the expression of extracellular matrix proteins^20,21^. However, these deactivated HSCs are distinct from quiescent HSCs in their ability to more quickly re-activate in response of a liver injury to bring about a stronger fibrotic response after the second insult^20,21^, effectively priming them for the second hit. Given that the major signals that activate HSCs *in vitro* and *in vivo* are proinflammatory cytokines, and that exposure to GWI-related chemicals results in a persistent low-level upregulation of these cytokines, it is conceivable that in GWI, HSCs are primed and will react more strongly to chronic liver injury.

In this study we aimed to determine if the exposure to GWI-related chemicals and stress is conducive to changes in cholangiocyte proliferation, proinflammatory immune response, HSC activation and liver fibrosis in an animal model of cholestasis, i.e. bile duct ligation (BDL), when compared to GWI unexposed counterparts. We considered the “two-hit” hypothesis to explore the possibility that exposure to Gulf War chemicals and stress environment could sensitize the liver and prime it for a stronger than normal immune response and hepatic fibrosis in case of a chronic disease such as biliary cholestasis. It has been established that the underlining mechanisms of several hepatic dysfunctions, e.g., non-alcohol fatty liver disease (NAFLD), can be explained by the “two hits” or even “multiple hits” hypothesis. According to these, subtle but significant changes in the liver, such as lipid accumulation in the case of NAFLD due to high fat diet and sedentary lifestyle for example, act as a first insult of the liver with no evident symptoms of disease; in the event of a second liver insult such as an acute or chronic hepatic disease, the inflammation and fibrosis of the liver are enhanced in the sensitized liver due to molecular and cellular events from the first insult^22,23^.

## Results

### Pre-exposure to GWI-related chemicals and stress increases serum markers of liver injury and hepatic necrosis in BDL-challenged rats

**Figure 1:**
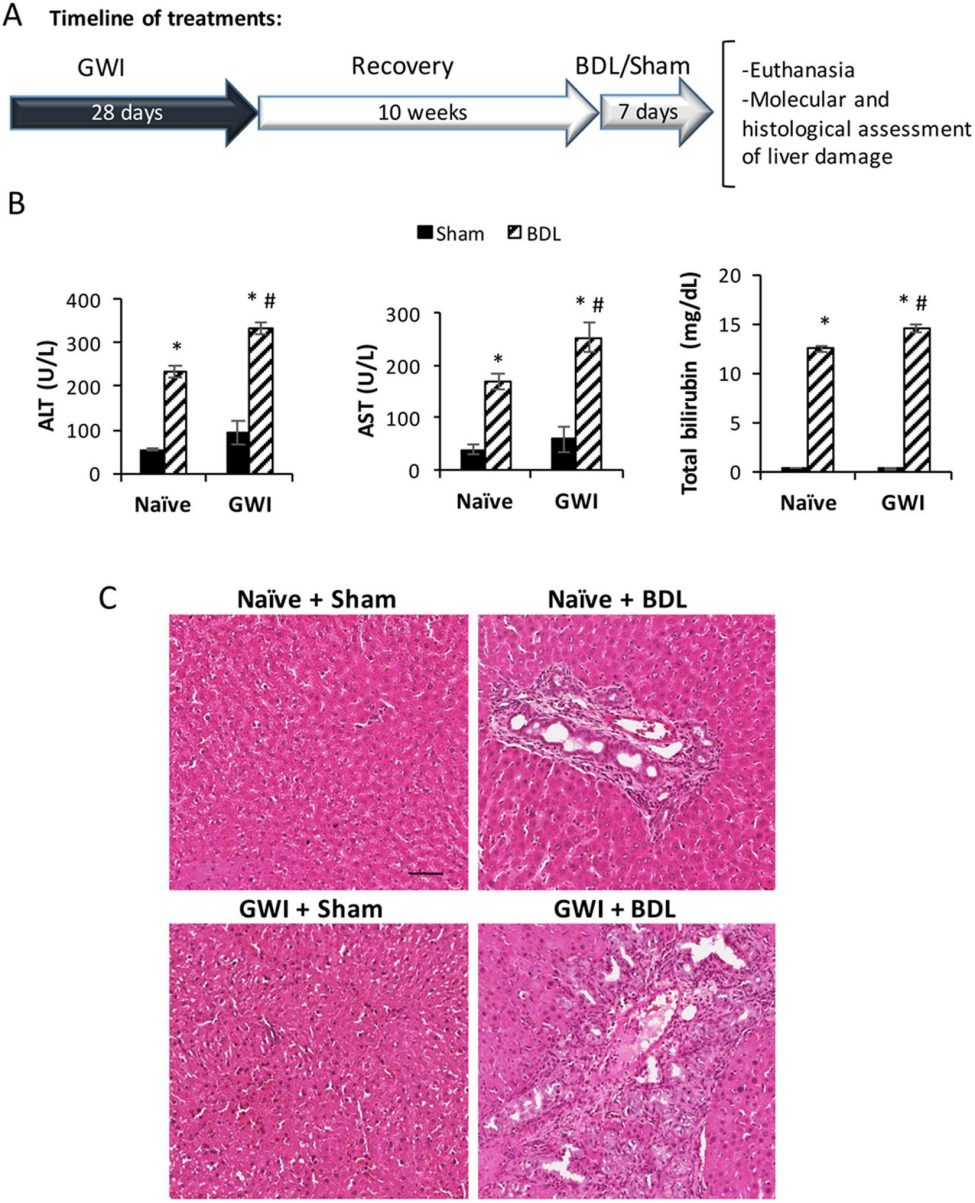
Timeline of treatments with Gulf War Illness (GWI)-related chemicals/stress and bile duct ligation (BDL), and serum markers of liver damage. (**A**) Diagram of treatments timeline. (**B**) Alanine aminotransferase (ALT), aspartate aminotransferase (AST) and total bilirubin were assessed in serum of GWI or naïve (unexposed) rats which were subject to BDL or sham surgery. p < 0.05. * BDL vs Sham; #, GWI vs naïve. (**C**) Hematoxylin and eosin (H&E) staining of liver sections from naïve and GWI-chemical exposed rats that underwent sham or BDL surgeries. Scale bar, 100 μM.

In order to assess a possible influence of GWI-related chemicals and stress on the liver function in healthy versus cholestatic animals, male Sprague Dawley rats were subjected to the treatment regimen shown in Fig. 1A. A group of animals denoted GWI were treated with a combination of nerve gas prophylactic drug PB and two types of insecticides, DEET and permethrin, followed by restraint stress to mimic the Gulf War environment, daily for 28 days. Ten weeks after the cessation of exposure to the chemical and stress treatment, the GWI rats underwent either BDL or sham surgery. Control groups consisting of age-matched male rats were not treated with chemicals but underwent either BDL or sham surgery and are referred to as naïve. Hepatic function biomarkers alanine aminotransferase (ALT), aspartate aminotransferase (AST) and total bilirubin were assessed in serum of animals seven days after BDL or sham surgeries. As shown in Fig. 1B, these serum markers of liver damage were increased by BDL in both naïve and GWI rats. Significantly, higher levels of ALT, AST and total bilirubin were found in sera from GWI-treated animals than in naïve controls after BDL surgery. Conversely, there were no differences between GWI and naïve rats that underwent sham surgery. When histological changes were assessed by H&E staining of the livers, it was confirmed that rats exposed to GWI-related chemicals and stress exhibited a greater degree of biliary injury than the naïve rats after BDL-induced cholestasis (Fig. 1C). These data suggest that the animals treated only with GWI-related chemicals and stress had no signs of liver injury. However, when hepatic cholestasis was induced by BDL surgery, the rats subjected to GWI-related exposure manifested worse damage of the liver than the naïve, unexposed animals.

### Pre-exposure to GWI-related chemicals and stress enhances BDL-induced enlargement of intrahepatic bile duct mass (IBDM)

**Figure 2:**
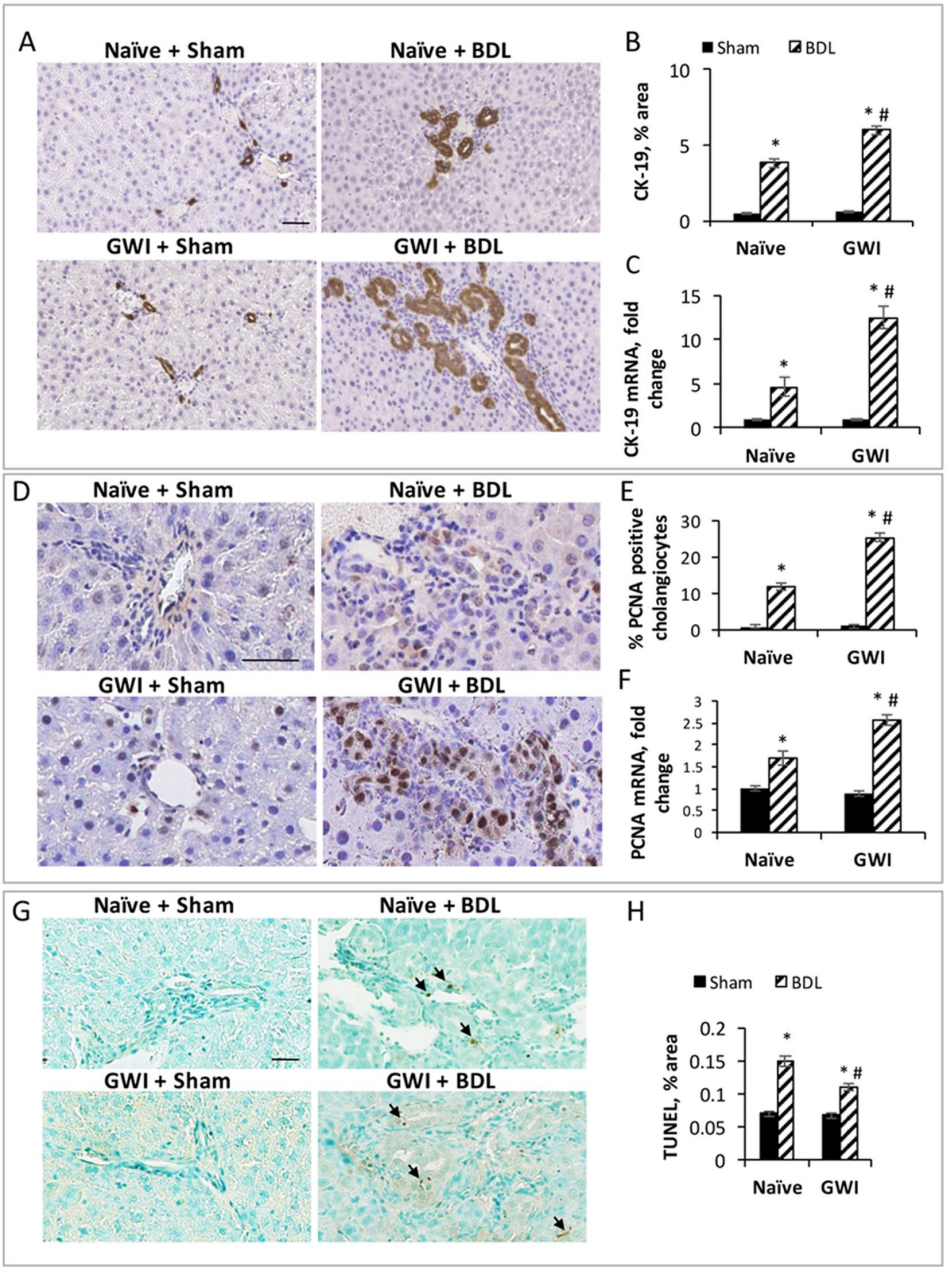
Measurement of intrahepatic bile duct mass (IBDM), cholangiocyte proliferation and apoptosis in GWI versus naïve rats that underwent sham or BDL surgeries. (**A**) Images of immunohistochemistry (IHC) for cholangiocyte marker cytokeratin 19 (CK-19). (**B**) Plot of data from image analysis: percent pixel area per field stained for CK-19 was measured for all four animal groups. (**C**) Plot of RT-qPCR data for CK-19 in the liver of GWI with sham or BDL versus naïve animals with sham or BDL surgeries. (**D**) IHC images of proliferating cell nuclear antigen (PCNA) in livers of GWI-treated animals that had sham or BDL surgeries as compared to naïve animals subjected to sham or BDL. Quantification of PCNA IHC images are shown in (**E**). (**F**) RT-qPCR of PCNA marker in livers of GWI and naïve rats subjected to sham or BDL surgeries. (**G**) Images of apoptotic cells (stained in brown) versus normal cells counterstained with methyl-green, in liver sections of GWI and naïve animals which had sham or BDL surgeries. Quantification of apoptotic cells in all groups is shown in (**H**). p < 0.05. * BDL vs Sham; #, GWI vs naïve. Scale bar, 100 μM.

A key feature of cholestatic liver injury caused by BDL is the expansion of IBDM. The amount of ductal mass was determined by immunohistochemistry (IHC) of cytokeratin 19 (CK-19), a marker specific for cholangiocytes, the cells lining the bile ducts. IHC images and quantifications of CK-19-positive cells in the livers from GWI or naïve animals that underwent BDL or sham surgery are shown in Fig. 2A,B. The results demonstrate that the increase in IBDM was much larger in GWI rats than in naïve controls after BDL. Specifically, a 36% increase in IBDM after BDL surgery was observed in cholestatic animals which had been exposed to GWI-related chemicals as compared to naïve animals. CK-19 expression at the mRNA level was also assessed (Fig. 2C) and found to be increased 2.3-fold in the livers of GWI-treated animals than in naïve controls after BDL-induced cholestasis.

The increase in IBDM observed after BDL is a result of the balance between cholangiocyte proliferation and apoptosis. In order to assess the proliferation of cholangiocytes, we performed IHC for proliferating cell nuclear antigen (PCNA) (Fig. 2D). The IHC images were then analyzed to find the percentage of cholangiocytes expressing PCNA (Fig. 2E). There was a 2-fold increase in proliferating cholangiocytes in the livers from cholestatic GWI rats than in cholestatic naïve animals. PCNA mRNA expression was also significantly elevated in GWI-BDL rats as compared to naïve BDL control rats (Fig. 2F).

To determine a possible role for apoptosis in the dramatic change of IBDM in GWI versus naïve cholestatic rats, we ran a Terminal deoxynucleotidyl Transferase (TdT) dUTP Nick-End Labeling (TUNEL) assay on liver sections from all treatments and control groups. As shown in Fig. 2G,H, very few apoptotic cells were detected in livers of rats with sham surgery in either GWI or naïve group. Apoptotic cells within or around biliary ducts were detected mostly in BDL-operated animals, both GWI and naïve. Interestingly, there was a smaller amount of apoptotic cholangiocytes in GWI rats than in naïve BDL-rats, suggesting that the ability of the liver to activate apoptosis in order to counteract the abnormally high proliferation of cholangiocytes, was diminished in GWI-exposed animals.

### Exposure to GWI-related chemicals and stress before BDL-induced cholestasis enhances the expression of proinflammatory cytokines

**Figure 3:**
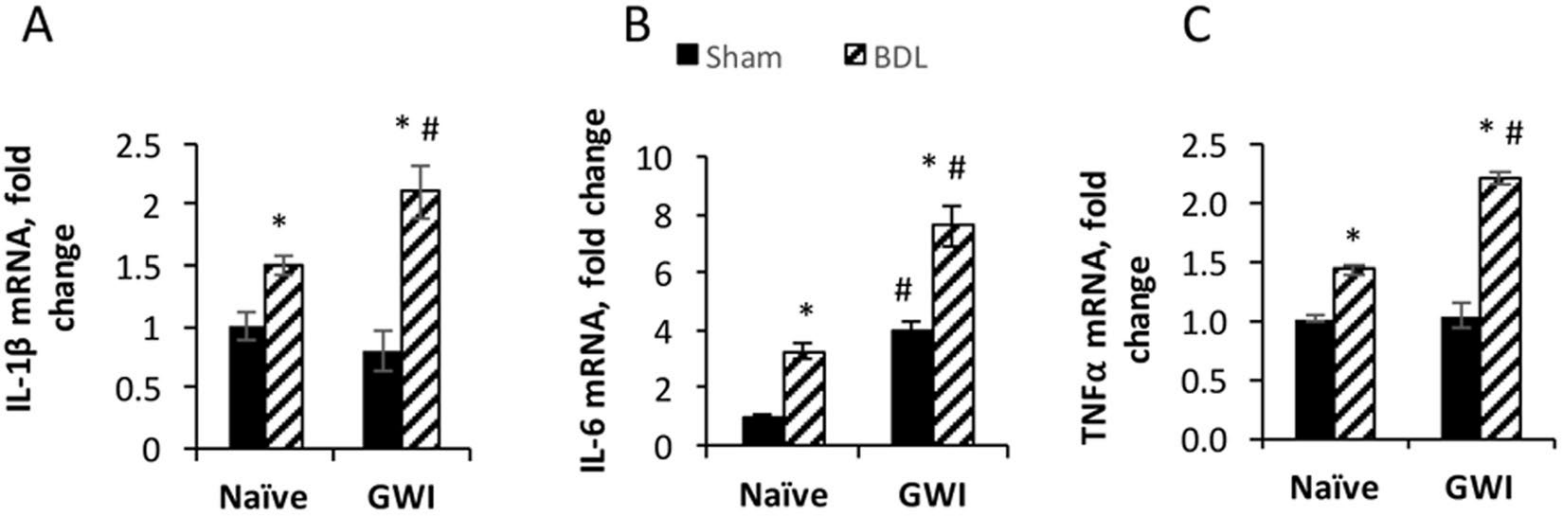
Proinflammatory cytokines in the livers of GWI versus naïve rats subjected to sham or BDL surgery. Expression of: (**A**) interleukin-1 (IL-1β); (**B**) interleukin-6 (IL-6); and (**C**) tumor necrosis factor–alpha (TNFα) was determined by RT-qPCR assays of RNA isolated from the livers of GWI and naïve animals subjected to sham or BDL surgeries.

Inflammation is associated with hepatic cholestasis^24,25^, so we assessed the effect of GWI-related chemicals on the expression of several proinflammatory cytokines in GWI and naïve rats that underwent sham or BDL surgery (Fig. 3). As expected, the mRNA levels of IL-1β, IL-6 and TNFα were higher in BDL compared to sham-operated rats, regardless of exposure to GWI-related chemicals. All three proinflammatory cytokines were more abundant in livers from BDL-operated animals that had been exposed to GWI-related chemicals as compared to naïve controls. Interestingly, IL-6 was found to be higher in GWI than naïve rats even in the absence of cholestasis in animals that underwent sham surgeries. These data suggest that the GWI-related chemicals affect the hepatic immunity of animals in our experimental model, raising the level of inflammation of the liver, especially after BDL-induced cholestasis.

### Pre-exposure to GWI-related chemicals sensitizes the liver to increased recruitment of monocytes/macrophages after BDL-induced cholestasis

**Figure 4:**
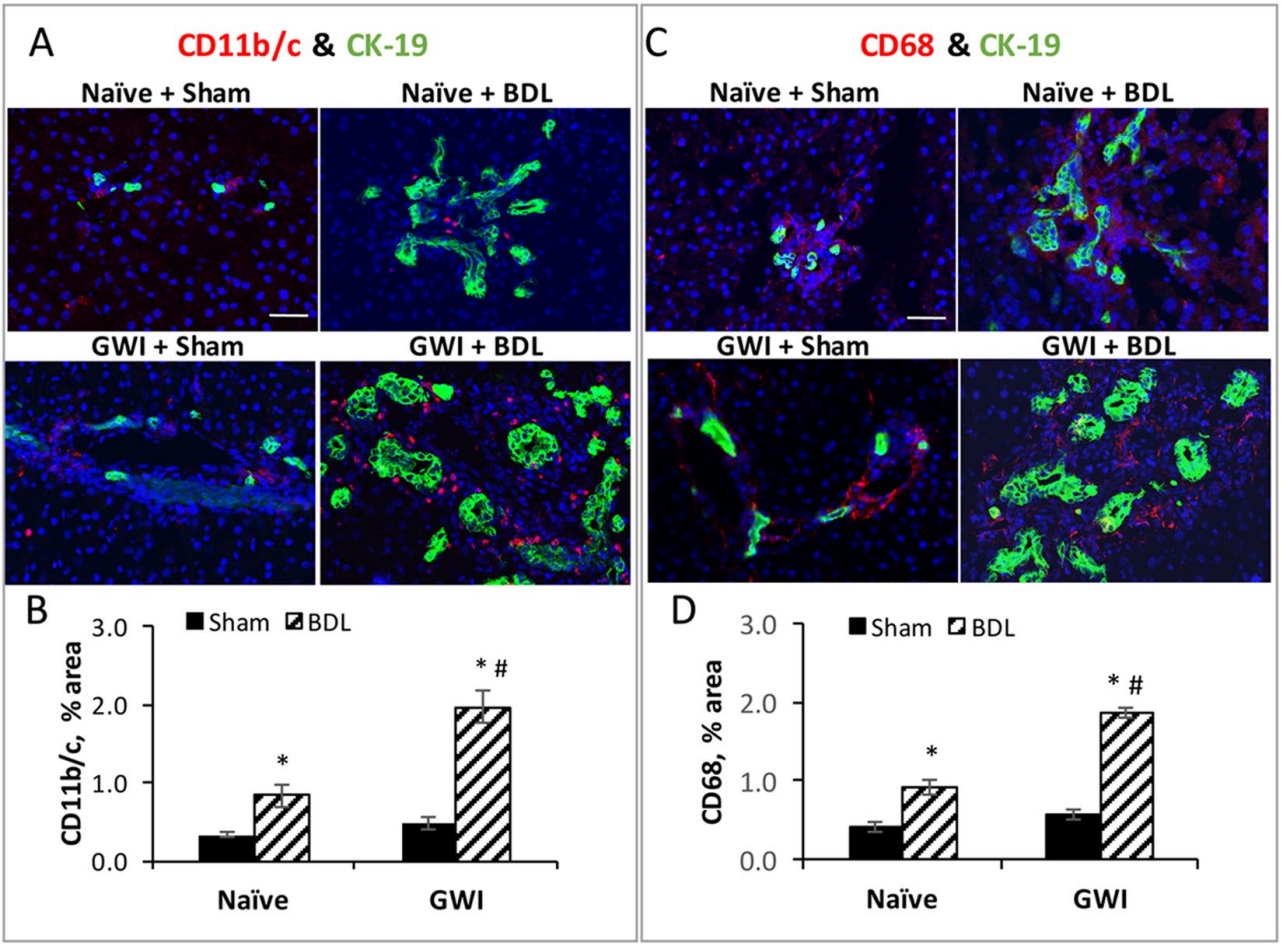
Density and localization of CD11b/c^+^ and CD68^+^ macrophages in the liver of GWI and naïve rats that underwent sham or BDL surgeries. (**A**) Immunofluorescence (IF) of CD11b/c^+^ macrophages (red) and cholangiocytes (green) versus nuclei (stained in DAPI, shown in blue). (**B**) Plot of image analysis data, showing the percentage of pixel area per field measured for CD11b/c positive cells. (**C**) Same as in (**A**) except that in red are shown CD68^+^ cells. (**D**) Plot of data from image analysis, showing the percentage of pixels per field for CD68^+^ cells. Scale bar, 100 μM.

Based on finding increased proinflammatory cytokines in the livers of GWI-rats as compared to naïve controls, we investigated whether there were any changes in the amount of several types of macrophages as a result of exposure of the animals to GWI-related chemicals before sham or BDL procedure. Thus, CD11b/c-, CD68- and F4/80-expressing macrophages were detected and quantified by IHC (Suppl. Fig. 1). All of these macrophage populations were found in higher numbers in GWI rats as compared to naïve controls when subjected to BDL surgery. CD11b/c^+^ cells in particular were slightly increased even in the livers of GWI rats subjected to sham, as compared to naïve controls (Suppl. Fig. 1). Unlike CD68-positive cells which were uniformly distributed throughout the liver, CD11b/c macrophages in naïve and GWI rats with cholestatic livers were in close proximity to bile ducts (Suppl. Fig. 1). Double immunofluorescence (IF) labeling of CD11b/c^+^ macrophages and CK-19-expressing cholangiocytes (Fig. 4A) indicated that these types of monocytes/macrophages were strongly recruited around highly proliferating cholangiocytes after BDL-induced cholestasis in both naïve and GWI rats. The amount of CD11b/c^+^ macrophages in the livers of GWI rats which underwent BDL was 2.5 fold higher than in naïve rats which had the same surgery (Fig. 4B), indicating that the exposure to GWI-related chemicals predisposed the rats to recruit more of these cells to their liver than the unexposed controls. Even in the groups of rats that underwent sham surgeries, there was a trend of increased CD11b/c^+^ macrophages in GWI rats than in naïve controls, though significance was not reached (Fig. 4B, Suppl. Fig. 1).

Immunofluorescence assays of CD68^+^ macrophages and CK-19-expressing cholangiocytes in the livers of naïve and GWI rats subjected to sham or BDL surgeries demonstrated a significant increase in the amount of these macrophages after BDL-induced cholestasis (Fig. 4C,D). A larger increase was detected in GWI than in naïve rats. However, unlike CD11b/c^+^ cells, the CD68^+^ cells were distributed close to the blood vessels rather than around the bile ducts.

### Most macrophages recruited to the livers of rats exposed to GWI-chemicals and stress were CD11b/c^+^F4/80^−^CD68^−^ M1 proinflammatory macrophages

**Figure 5:**
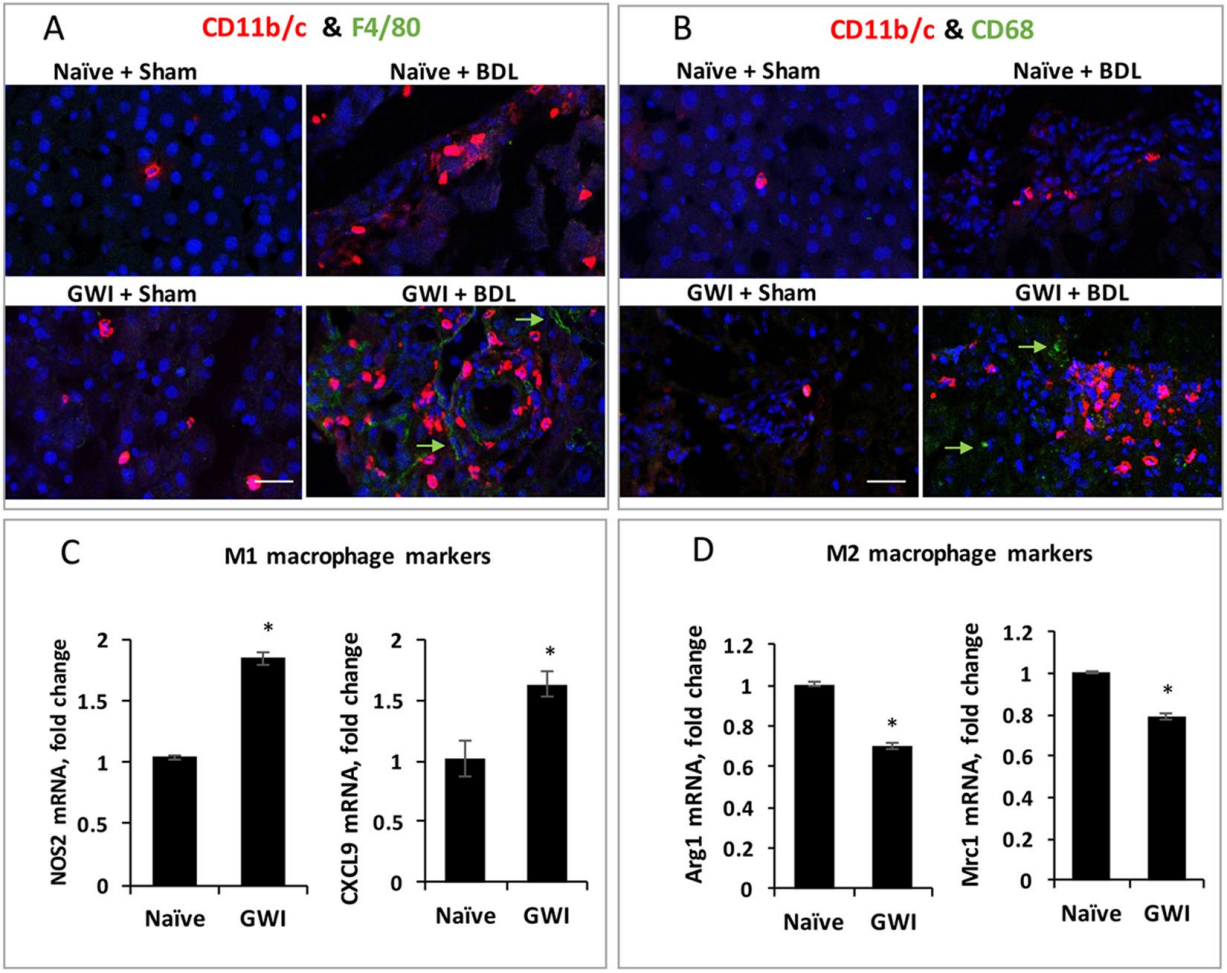
Characterization of specific subgroups of Cd11b/c^+^ macrophages in the livers of GWI and naïve rats that underwent sham or BDL surgeries. Double labeling IF was performed in order to test the colocalization of CD11b/c^+^ with F4/80^+^ cells (**A**) and of CD11b/c^+^ with CD68^+^ cells (**B**). In red are shown CD11b/c^+^ cells in both (**A**,**B**) panels; in green are F4/80^+^ macrophages (**A**) or CD68^+^ macrophages (**B**). Nuclei were stained with DAPI and shown in blue in all images. (**C**) Results of RT-qPCR assays of M1 macrophages (**C**) and M2 macrophages (**D**) are shown for the following markers: NOS2 and CXCL9 in (**C**); Arg1 and Mrc2 in (**D**). The RNA was isolated from CD11b/c-positive cells which were fluorescence immuno-labeled and cut off the liver sections by laser capture microdissection. Scale bar, 100 μM.

We further investigated the subgroups of CD11b/c^+^ macrophages, and determined the colocalization of CD11b/c with F4/80 and CD68 macrophage markers. As shown in Fig. 5A, the F4/80^+^ cells were less frequently detected than the CD11b/c^+^ macrophages, and there was no colocalization of the two markers in any cells, suggesting that the CD11b/c^+^ cells recruited in the liver as a result of cholestasis were CD11b/c^+^F4/80^−^ macrophages. When the colocalization of CD11b/c marker with CD68 was tested (Fig. 5B), there was no overlapping of the two markers in the same cells, indicating that the CD11b/c^+^ macrophages did not express CD68 marker.

Many published studies demonstrated that once recruited into the liver, monocytes start to differentiate and polarize into proinflammatory M1 and anti-inflammatory M2 phenotypes^26^. We investigated this nature of CD11b/c^+^ macrophages by labeling these cells with CD11b/c antibody conjugated to a fluorescent dye, followed by laser capture microdissection (LCM). The collected cells were then used for the isolation of total RNA, synthesis of cDNAs and RT-qPCR for markers of M1 and M2 macrophages (Fig. 5C,D). NOS2 and CXCL9, markers of M1 macrophages, were found to be significantly increased while Arg1 and Mrc1, markers of M2 macrophages, were decreased in CD11b/c^+^ macrophages from rats that had been exposed to GWI chemicals as compared to naïve controls when both these groups underwent sham surgery. These data demonstrate that even without challenging the liver with a cholestatic injury, in sham-operated animals the CD11b/c^+^ cells tended to be polarized toward having more proinflammatory and less anti-inflammatory characteristics. This itself can be one of the causes of an enhanced immune response upon BDL-induced cholestasis in GWI rats as compared to naïve controls.

### Hepatic stellate cells (HSCs) activation and proliferation is increased in animals with BDL-induced cholestasis due to pre-exposure to GWI-related treatment

**Figure 6:**
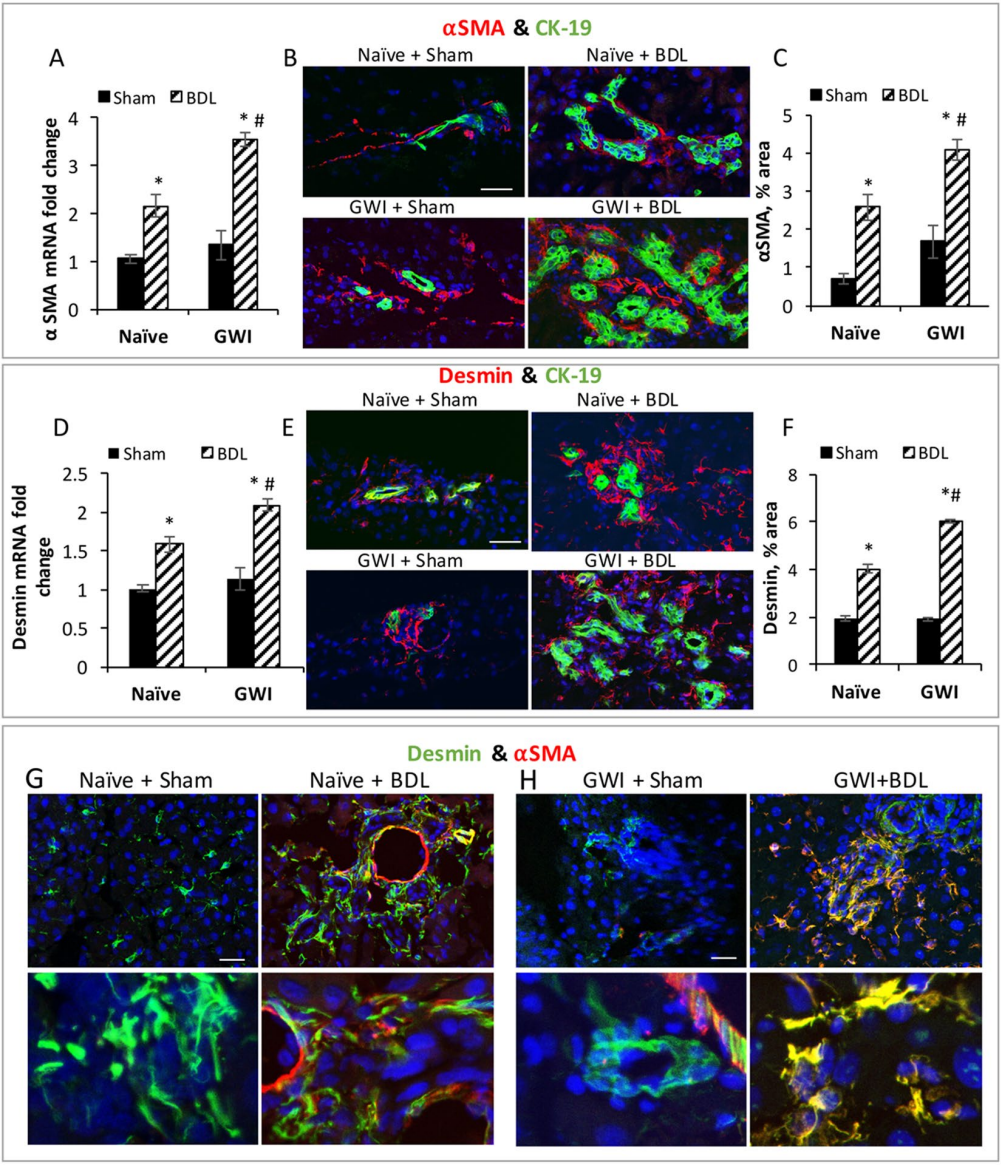
Activation and proliferation of hepatic stellate (HSC) cells in GWI and naïve rats that underwent sham or BDL surgery. (**A**) Plot of data from RT-qPCR assays of alpha-smooth muscle actin (αSMA) in the livers of GWI and Naïve animals subjected to sham or BDL procedure. (**B**) IF of αSMA (red), CK-19 (green); cell nuclei were counterstained with DAPI (blue). (**C**) Plot of data from image analysis of αSMA expression in percent pixel area per field. (**D**) RT-qPCR results for desmin in livers of GWI and naïve rats that underwent sham or BDL surgery. (**E**) IF of desmin (red) and cholangiocytes (green) in liver sections of GWI and naïve rats with sham or BDL surgeries. Cell nuclei were counterstained with DAPI and shown in blue. (**F**) Plot of data from IF image analysis showing changes in desmin protein in the livers of various treatment groups. Desmin amount was expressed as percentage of pixel area per field. p < 0.05. * BDL vs Sham; #, GWI vs naïve. (**G**) Confocal microscopy colocalization of αSMA and desmin in livers of GWI and naïve rats when subjected to sham or BDL surgeries. Scale bar, 100 μM.

Because activation of HSCs is a critical event in the process of fibrogenesis in the cholestatic liver^27^, we assessed the expression of alpha smooth muscle actin (αSMA), a marker of activated HSCs, in livers from naïve and GWI rats that underwent sham or BDL surgery (Fig. 6A–C). αSMA mRNA was expressed more in GWI animals than in naïve controls after BDL surgeries. We then estimated the expression level of αSMA protein by IF and image analysis as illustrated in Fig. 6B,C. αSMA protein was more abundant in the livers of GWI rats than in naïve controls after BDL surgery. These results suggest that GWI animals have a higher number of activated HSCs than naïve animals when challenged with BDL surgery. These results were confirmed by IHC assays (Suppl. Fig. 2).

We also estimated the total amount of HSCs, including activated, inactivated and quiescent HSCs by assessing the expression of desmin, a protein characteristic for these cells (Fig. 6D–F). Desmin mRNA expression was significantly higher in livers of GWI than naïve rats when challenged with BDL surgery, but not with sham operation (Fig. 6D). The expression of desmin protein was assessed by IF, and a dual immunostaining of desmin and CK-19 was used to show the distribution of HSCs in relation to cholangiocytes (Fig. 6E). Desmin-expressing cells were detected mostly around CK-19-stained ductal structures in the liver of sham or BDL-challenged animals either GWI or naïve (Fig. 6E). The amount of desmin-expressing HSCs was significantly increased by BDL surgery in naive and even more so in GWI animals (Fig. 6F). In summary, the number of HSCs was demonstrated to be increased by GWI-related chemical exposure prior to BDL-induced cholestasis.

We also used confocal microscopy colocalization of αSMA and desmin to determine the level of HSC activation in GWI animals as compared to naïve controls (Fig. 6G,H). The livers of both naïve and GWI groups that underwent sham surgery had only a small number of HSCs which were quiescent or inactivated, expressing desmin (detected in green) but not αSMA (in red). In the livers of rats with BDL surgery however, many HSCs were activated and detected in yellow pseudocolor, indicative of overlapping of desmin and αSMA in the same cells. Interestingly, even though there was a high increase in the total HSC number after BDL in both naïve and GWI-treated rats, there were more activated HSCs in GWI than in naïve group (right side pictures in panel H versus right side pictures in panel G, Fig. 6).

### GWI-related chemicals and stress pre-exposure increases liver fibrosis after BDL-induced cholestasis

**Figure 7:**
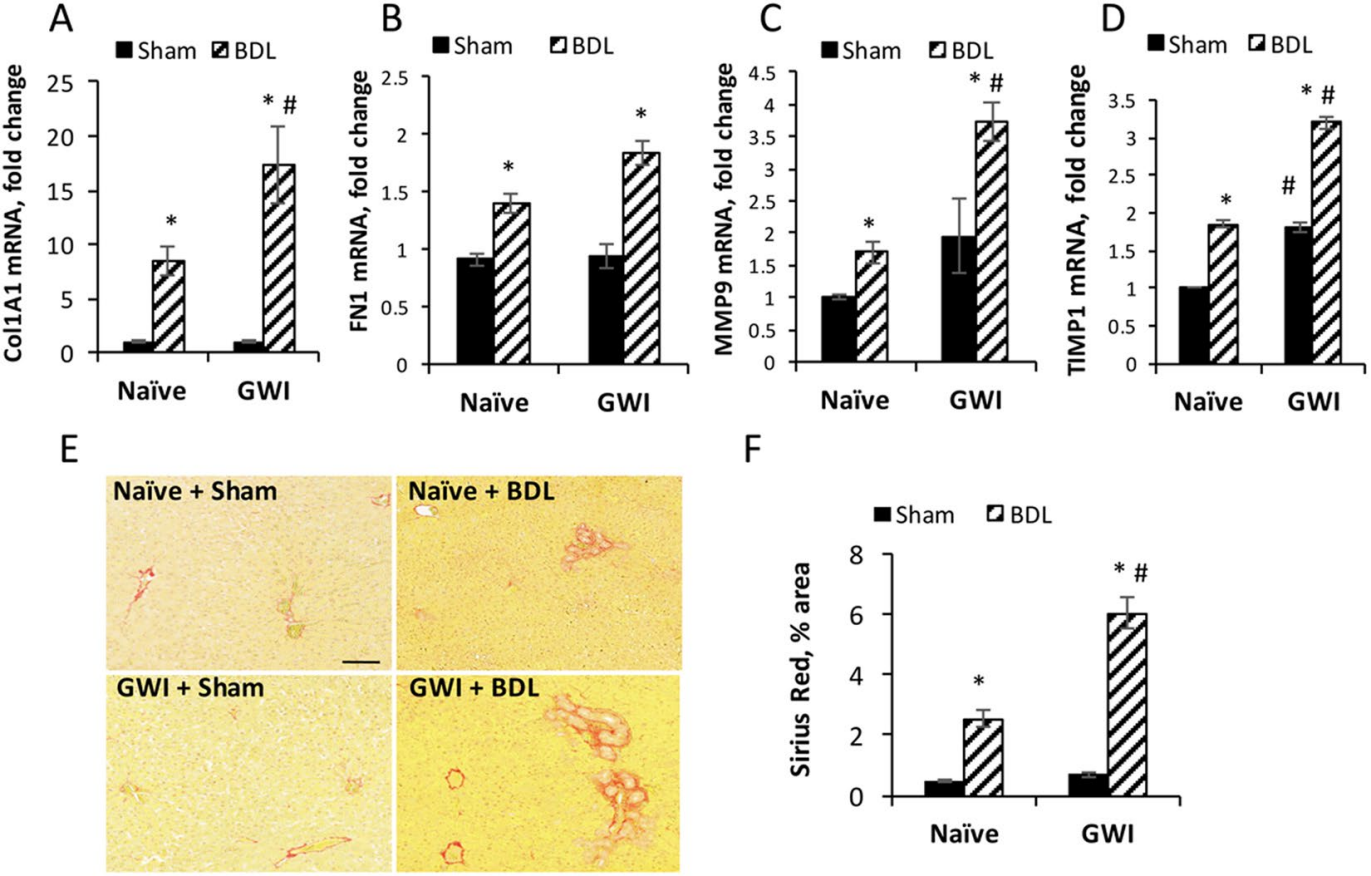
Hepatic fibrosis in GWI-chemicals treated rats as compared to naïve controls that underwent sham or BDL surgeries. (**A**–**D**) Plots of RT-qPCR data for markers of hepatic fibrosis: collagen 1A1(Col1A1), fibronectin 1 (FN1), matrix metalloproteinase 9 (MMP9), tissue inhibitor of metalloproteinases 1(TIMP1). (**E**) Images of liver sections stained with Sirius Red, a dye which specifically stains collagen I and collagen III fibrillary structures. (**F**) Plot of data from image analysis of Sirius Red staining of liver sections. p < 0.05. * BDL vs Sham; #, GWI vs Naïve. Scale bar, 100 μM.

We investigated differences in liver fibrosis markers in GWI compared to naïve animals when undergoing sham or BDL surgeries (Fig. 7). RT-qPCR assays of hepatic fibrosis markers such as collagen type1A1 (Col1A1), fibronectin1 (FN1), matrix metalloproteinase-9 (MMP9) and tissue inhibitor of metalloproteinase 1 (TIMP1) demonstrated that all these markers were more abundant in livers of GWI rats than naïve controls when challenged with BDL as compared to sham surgeries. Moreover, one of these markers, TIMP1, was found to have a higher expression level in GWIs than naïve controls in sham-operated animals, suggesting that some changes in fibrogenesis markers can occur with exposure to GWI-related chemicals and stress alone.

A usual assay for assessing liver fibrosis is Sirius Red staining of collagen types I and III, which are increased in hepatic fibrosis^28^. Our results of Sirius Red staining of liver sections from GWI versus naïve rats that underwent BDL or sham surgeries are shown in Fig. 7E,F. All animals subjected to sham operations, regardless if they had been exposed to GWI-related chemicals or not, exhibited a very low level of Sirius Red-stained fibers, suggesting that the treatment with GWI-related chemicals did not cause any liver fibrosis. However, when animals were challenged with BDL, they developed larger fibrotic areas than sham-operated animals; those which had been exposed to GWI-related chemicals exhibited 2.5-fold more fibrosis than their naïve counterparts (Fig. 7F). Overall, these data demonstrate that exposure of animals to GWI-related chemicals and stress before BDL surgery caused a significant aggravation of the liver fibrosis after BDL-induced cholestasis.

## Discussion

The aim of this study was to test in an animal model the possibility that a combination of chemicals similar to those used during the Persian Gulf War in 1990–91, including the nerve gas prophylactic drug PB and insecticides DEET and permethrin, in addition to stress, may affect the hepatic immune response and cause a significant aggravation of a subsequent cholestatic liver injury and fibrosis. Our experiments demonstrated that in GWI rats that underwent sham surgeries, the hepatic serum markers were in the normal range and similar to naïve controls with sham surgeries, indicating that the treatment with GWI-related chemicals did not affect liver functions. However, when hepatic cholestasis was induced by BDL, all three parameters ALT, AST and total bilirubin were more elevated in the GWI group than in naïve controls.

Quantification of IBDM in our experimental model demonstrated a remarkable increase in the ductal mass caused by exposure to GWI-related compounds and stress prior to BDL-induced cholestasis. A cell proliferation marker, PCNA, was found to be expressed more in cholangiocytes of GWI than in naïve controls after BDL surgery, supporting a higher proliferation rate of these cells in cholestatic liver after exposure to GWI-related chemicals and stress. Apoptosis assays demonstrated that there were very few apoptotic cells overall, and particularly within the bile duct areas, in GWI and naïve animals after sham surgery, but significantly more apoptotic cells were observed after BDL. Interestingly, GWI treatment reduced the hepatic apoptosis induced by BDL, suggesting that the ability of the liver to counteract the abnormal proliferation of cholangiocytes due to BDL by apoptotic processes was diminished after exposure of the animals to GWI-related chemicals/stress. To our knowledge, these are the first data to demonstrate a larger IBDM and increased proliferation of cholangiocytes as a result of exposure to GWI-related compounds prior to BDL-induced cholestasis in an experimental model. Future clinical studies on cholangiopathies and other liver diseases in veterans with GWI symptoms may confirm these results.

When assessing the level of proinflammatory cytokines such as IL-1β, IL-6 and TNFα in the livers of GWI versus naïve animals, we determined that all these cytokines were increased in GWI as compared to controls after BDL. Interestingly, IL-6 was found to be elevated in GWI as compared to naïve in the groups subjected to sham surgery, suggesting that this particular cytokine was abnormally expressed due to only GWI-related chemical treatment, without cholestasis challenge. Several clinical studies reported elevated markers of inflammation in veterans with GWI symptoms. Thus, elevated platelet count, C-reactive protein and thromboxane analog-induced platelet aggregation suggested increased vascular inflammation in veterans suffering from GWI^29^ even though the results of this pilot study were not fully confirmed in a larger study^16^. A study that measured the levels of several cytokines in GWI-affected subjects found TNFα to be among the most significant cytokines associated with Gulf War syndrome^15^. The results in our experimental model showing that TNFα expression was significantly increased in GWI-treated animals as compared to unexposed ones after BDL-induced cholestasis are in line with findings in human studies. Another interesting study monitored daily several cytokines in veterans suffering from GWI symptoms and in healthy veterans as controls, and concluded that higher fatigue severity days were associated with greater IL-1β and IL-15^30^. These data are consistent with results from our GWI experimental model, suggesting that under stress conditions, such as extraneous fatigue or liver disease, IL-1β among other cytokines can reach levels higher than normal due to prior exposure to GWI-related chemicals.

The role of hepatic inflammation in various liver diseases is well-documented^26,31,32^. Hepatic macrophages especially play a critical role in the pathogenesis of chronic liver diseases which result in increased inflammation and fibrosis. Liver macrophages are a heterogeneous population of immune cells emerging either from resident Kupffer cells or by recruitment of monocytes/macrophages from blood flow in response to chemokines secreted upon hepatic injury^32,33^. In this study we focused on macrophages expressing CD11b/c, CD68 or F4/80 markers. While CD11b/c is recognized as a marker of recruited monocytes/macrophages, it is still debated whether CD68 is an absolute marker of Kupffer resident cells^34,35^. A possible effect of GWI-related chemicals and stress on liver macrophage functions in relation to subsequent and unrelated biliary or liver diseases has not been investigated so far in clinical or animal studies. In our experimental model, exposure to GWI chemicals and stress before BDL surgery resulted in an increased number of all tested macrophages, in particular CD11b/c^+^ and CD68^+^ hepatic macrophages. The number of CD11b/c^+^ macrophages increased even with GWI treatment alone. We further established that these CD11b/c^+^ macrophages did not colocalize with CD68, nor F4/80-expressing cells. The CD68^+^ and F4/80^+^ macrophages were sparser as compared to CD11b/c^+^ cells. In contrast, CD11b/c^+^ macrophages were very abundant and in close proximity to proliferating bile ducts. Our findings point to an important role of CD11b/c^+^ macrophages in GWI-chemical and stress exposure, increasing inflammation and fibrogenesis after BDL and cholestasis. These results are consistent with previously published reports on the role of CD11b/c^+^ macrophages in fibrogenesis. For example, it was demonstrated that CD11b/c^+^ macrophage deletion in mice resulted in amelioration of hepatic fibrosis^36^. We also demonstrated that treatment with GWI-related chemicals and stress alone, without BDL challenge, resulted in CD11b/c^+^ macrophages being more polarized toward M1 proinflammatory and less toward M2 anti-inflammatory type. These changes may explain the enhanced immune response of GWI-rats as compared to naïve controls after undergoing BDL surgery. M1 macrophages are known to have pro-inflammatory function by secreting TNFα and IL-6 cytokines, while M2 macrophages emerge during tissue secreting anti-inflammatory cytokines such as IL-10^33^.

During fibrosis progression, monocyte-derived macrophages release cytokines perpetuating chronic inflammation and activating HSCs^37–39^. Activated HSCs undergo proliferation and transdifferentiation into collagen-producing myofibroblasts^36,40^. In our experimental model of GWI, the assessment of GWI-related effects on HSCs demonstrated that exposure to GWI-compounds and stress prior to BDL-induced cholestasis caused a significant increase in activated HSCs expressing αSMA and more proliferation of all HSCs detected as desmin-positive cells. The change in HSC activation along with a low but significant proinflammatory status of the liver due to exposure to GWI-related chemicals explain, at least partly, the enhanced hepatic fibrosis detected in the livers of GWI-BDL rats as compared to naïve-BDL controls. Thus, the expression of many hepatic fibrosis markers such as Col1A1, FN1, MMP9 and TIMP1 was more abundant in GWI-BDL group than in naïve-BDL controls. Also, the Sirius Red-stained fiber structures of collagen I and III were much more numerous in GWI-BDL rats than in naïve-BDL animals. The fact that TIMP1 expression was significantly elevated, even in GWI-treated without BDL, is relevant for the possibility that GWI-related chemicals could cause changes in hepatic gene expression, with severe consequences during subsequent hepatic injuries. TIMP1, a potent inhibitor of matrix metalloproteinases, was reported to be upregulated during progressive fibrosis and downregulated during fibrosis reversal in humans and experimental models^41–43^. Its higher than normal expression in GWI animals could prevent metalloproteinases from degrading the increased amount of collagen, elastin and other extracellular matrix proteins produced by HSCs due to BDL-induced cholestasis in our animal model.

Overall, the results suggest that in our experimental model, exposure to GWI-related chemicals and stress alone, without BDL challenge, caused subtle but important changes, including: increased expression of proinflammatory cytokine IL-6 and higher number of M1 macrophages in the liver, decreased anti-inflammatory M2 macrophages, and increased expression of fibrotic markers. Moreover, after BDL-induced cholestasis, all tested hepatic parameters including liver function markers, proinflammatory cytokines, recruited monocytes/macrophages, activated HSCs and liver fibrosis markers were significantly enhanced in GWI animals compared to naïve controls. We can conclude that in our model, the exposure to GWI-related chemicals and restraint stress resulted in a low-level inflammation of the liver which, after cholestasis challenge, caused an increased inflammatory reaction, with very high recruitment of CD11b/c^+^F/80^−^CD68^−^ macrophages, enhanced proliferation of cholangiocytes, activation of HSCs, and ultimately increased liver fibrosis.

The data of this study on the effects of Gulf War-related chemicals on liver inflammation and fibrosis resulting from a subsequent hepatic injury in a rodent model may be relevant for humans. The Department of Veterans Affairs has published periodical reports on available current data on Gulf War service member population through the Gulf War Veterans Information System (GWVIS) at the va.gov website. A comprehensive study published in 2016 concluded that the only significant increased risk of mortality from cirrhosis of the liver in Army Gulf War veterans as compared to unexposed veterans, was in the case of exposure to nerve agents for more than two days in the Khamisiyah incident^44^. Studies on the actual predisposition for increased hepatic fibrosis in case of liver diseases after exposure to GWI-related chemicals in Gulf War veterans were difficult to accomplish, because even unexposed veterans have an increased incidence of cirrhosis and liver cancer due to high consumption of alcohol, smoking or drug use^44^.

The Department of Veterans Affairs (VA) has evaluated pesticide exposure alone and in combination with various prophylactic drugs such as PB as a possible cause of Gulf War Veterans’ chronic multisymptom illnesses^2^. The Research Advisory Committee on Gulf War Veterans’ Illnesses reports of 2004–2008 found evidence that chronic multisymptom illness is caused by exposure to PB and pesticides^2,45^. However, the chemical theory has been given less significance in recent volumes of the National Academy of Sciences series on Gulf War Illness^46,47^. The findings of our study on the influence of exposure to chemical compounds including DEET, permethrin and PB, on the inflammation and fibrosis of the liver in a rodent model of hepatic cholestasis are relevant not only in relation to GWI but also in a larger context. Our results suggest that in general, patients with hepatic cholestasis may suffer worse forms of liver inflammation and fibrosis if they had been exposed to chemicals such as those tested in our study. Epidemiologic studies involving veterans and agricultural workers suggest that pesticide-pesticide or pesticide-drug interactions may be related to GWI or elevated cancer risks, respectively^48,49^. Liver-mediated degradation and detoxification of certain insecticides such as permethrin was shown to be inhibited by DEET and metabolites of chlorpyrifos and other pesticides but not by PB^50,51^. The influence of combining various insecticides, pesticides and prophylactic or other types of drugs on metabolism and detoxification pathways in the liver, as well as on hepatic inflammation and possible priming for increased fibrosis in case of subsequent liver injuries, needs further investigation.

## Methods

All chemical reagents were purchased from Millipore Sigma-Aldrich (St. Louis, MO, USA) and were of the highest grade available, unless otherwise specified. DEET and permethrin were from ChemService Inc. (West Chester, PA). The antibodies used in IHC and IF experiments to detect CK-19, PCNA, CD11b/c, CD68, F4/80, αSMA and desmin were purchased from Abcam (Cambridge, MA, USA).

### Animal experiments

Male Sprague Dawley rats were purchased from Charles River (Worcester, MA) and maintained in a temperature-controlled environment at 20–22 °C with a 12:12 hours light-dark cycle, having free access to food and drinking water. All animal procedures were performed in accordance with the approval of the Baylor Scott & White Health Institutional Animal Care and Use Committee, (Protocol No: 2016-005). A group of 10 animals were administered a combination of chemicals consisting of PB (2 mg/kg/day by gavage), DEET (60 mg/kg/day, dermal application), and permethrin (0.2 mg/kg/day, dermal application) for 28 consecutive days. The chemical dosages were based on previously published data from studies on a rat model of GWI^52^, on a mouse model of GWI^53^ and also taking into account the dosages used during the Gulf War by participating veterans^53–55^. Even though PB was provided as 30 mg tablets and recommended to be taken every 8 hours for a week before or around the time of a nerve agent attack, the exact dosage taken by the veterans is unknown^55^. However, an estimated 1.6–2 mg/kg/day is considered to be within the range corresponding to PB consumption during the Gulf War^55^. Each daily chemical treatment was followed by 15 min of manual restrained stress administration, for the same period of 28 days. The chemical and stress-treated rats or GWI group were allowed to recover from the treatment for 10 weeks. In the same time, a control group of untreated, age-matched male rats (n = 10) or the naïve group, were housed in the same facility as the GWI group, but in separate cages. Afterwards, each group underwent BDL (n = 5 each for GWI and naïve groups) or sham (n = 5 each for GWI and naïve groups) surgeries as previously described. Seven days after the surgeries, all rats were sacrificed, and their blood and livers were collected. The animals were medically surveilled on a weekly basis before surgeries and a daily basis after surgeries. No health changes were reported during the chemical and stress treatment, nor during 10 weeks of recovery. The animal groups in this study included: GWI + sham (treated with GWI-related chemicals and subjected to sham surgery), GWI + BDL (treated with GWI-related chemicals and subjected to BDL surgery), naïve + sham (not exposed to chemicals, subjected to sham surgery) and naïve + BDL (not exposed to chemicals, subjected to BDL surgery).

### Assays of serum biochemical markers of liver injury

ALT, AST and total bilirubin were quantitatively determined by using a Catalyst One Chemistry Analyzer instrument from IDEXX Laboratory, Inc. (Westbrook, Maine), and the corresponding kits necessary for measuring the three markers of liver dysfunction.

### Assessment of cholangiocyte proliferation and apoptosis by IHC

In order to measure the amount of IBDM in the livers of rats unexposed or exposed to GWI-related chemicals and subjected to sham or BDL surgeries, we performed IHC on sections of paraffin-embedded livers. The liver sections were 4 μm thick and were obtained with a Leica microtome. Antibodies specific to CK-19 and PCNA were used for labeling cholangiocytes and proliferating cells, respectively, followed by staining using a VectaStain ABC kit from Vector Laboratories Inc. (Burlingame, CA, USA), as described^28^. The sections were then mounted on slides and scanned with a Leica SCN400 scanner, with 20× optical magnification. Screenshots with 10× digital magnification were taken and used for quantification of field-stained pixel percentage of CK19- or PCNA-positive cells. The images were analyzed using ImageJ software downloaded from the NIH website. Apoptotic cells in the liver paraffin sections were assessed using a TUNEL kit from Abcam (Cambridge, MA, USA) according to manufacturer’s instructions and were counterstained with methyl green.

### Assessment of liver histopathology

Hematoxylin and eosin (H&E) staining of liver sections was performed as described^28^. The degree of fibrosis in liver from various treatment groups was assessed by staining collagen I and III fibers with Sirius Red kit from IHC World (Ellicott City, MD) and then processing the slides in the same manner as for IHC analysis.

### Assays of mRNA expression of genes with role in inflammation and hepatic fibrogenesis

Changes in mRNA expression of CK-19, PCNA, IL-1β, IL-6, TNFα, αSMA, desmin, COL1A1, FN1, MMP9, TIMP1, NOS2, CXCL9, Arg1, Mrc1in rat livers were estimated by performing RT-qPCR. Total RNA was isolated from liver tissue of rats from groups of various treatments using the RNeasy kit from Qiagen (Germantown, MD, USA) according to the manufacturer’s instructions. The reverse transcription of RNAs was done with iScript RT Supermix from Bio-Rad (Hercules, CA, USA), and the real time PCR assay was performed with iTaq Universal SYBR Green Supermix from Bio-Rad; corresponding primers were obtained from SABiosciences-Qiagen. The amplification reactions were run with an AriaMx Real Time PCR system from Agilent Technologies (Santa Clara, CA, USA). The control gene was GAPDH, and the results were calculated and expressed as 2^−ΔΔCt^ or fold change of each gene as compared to GAPDH standard.

### Assessing the expression and cellular distribution of proteins with role in liver cholestasis, inflammation and fibrosis

In order to determine the presence of proteins of interest in the liver of animals subjected to various treatments, immunofluorescence (IF) labeling of frozen sections was used, followed by confocal microscopy, as described before^28^. In parallel, we determined the expression of proteins of interest by IHC of paraffin sections of livers by using the VectaStain ABC systems from Vector Labs (Burlingame, CA), as described before^28^. Quantitative determinations were performed by image analysis using ImageJ software. Macrophages were detected by using antibodies specific to CD11b/c, CD68 and F4/80, while HSCs were detected with αSMA and desmin antibodies, which were purchased from Abcam (Cambridge, MA, USA).

### Statistical analysis of data

Quantifications obtained in all experiments were statistically analyzed and presented as average of results and the corresponding standard error of the mean (SEM). When comparing two groups, the significance was calculated using the unpaired Student’s *t*-test. We also used one-way ANOVA followed by an appropriate post-hoc test on GraphPad Prism (San Diego, CA, USA). The statistical difference was considered significant for *p* values less than 0.05.

## Electronic supplementary material

Supplementary figures

## Data Availability

The datasets generated and analyzed during the current study are available from the corresponding author on reasonable request.
