# Supplementary material for: Gulf war illness-related chemicals increase CD11b/c+ monocyte infiltration into the liver and aggravate hepatic cholestasis in a rodent model
Source: Sci Rep. 2018 Sep 3;8:13147. doi: 10.1038/s41598-018-31599-9 (PMC6120951; doi:10.1038/s41598-018-31599-9)
Supplement: Supplementary file 1 — Supplementary figures [file 41598_2018_31599_MOESM1_ESM.pdf]

Gulf war illness-related chemicals increase CD11b/c$^+$ monocyte infiltration into the liver and aggravate hepatic cholestasis in a rodent model

Supplementary information

Anca D. Petrescu [1,2], Stephanie Grant [1,2], Gabriel Frampton [1,2], Matthew McMillin [2], Jessica Kain [1], Maheedhar Kodali [2,3], Ashok K. Shetty [2,3], Sharon DeMorrow [1,2]*

[1] Department of Medical Physiology, Texas A&M College of Medicine, Temple, 76504, USA
[2] Central Texas Veterans Healthcare System, Temple 76504, USA
[3] Institute for Regenerative Medicine, Department of Molecular and Cellular Medicine, Texas A&M College of Medicine, College Station, 77843, USA

[*]demorrow@medicine.tamhsc.edu

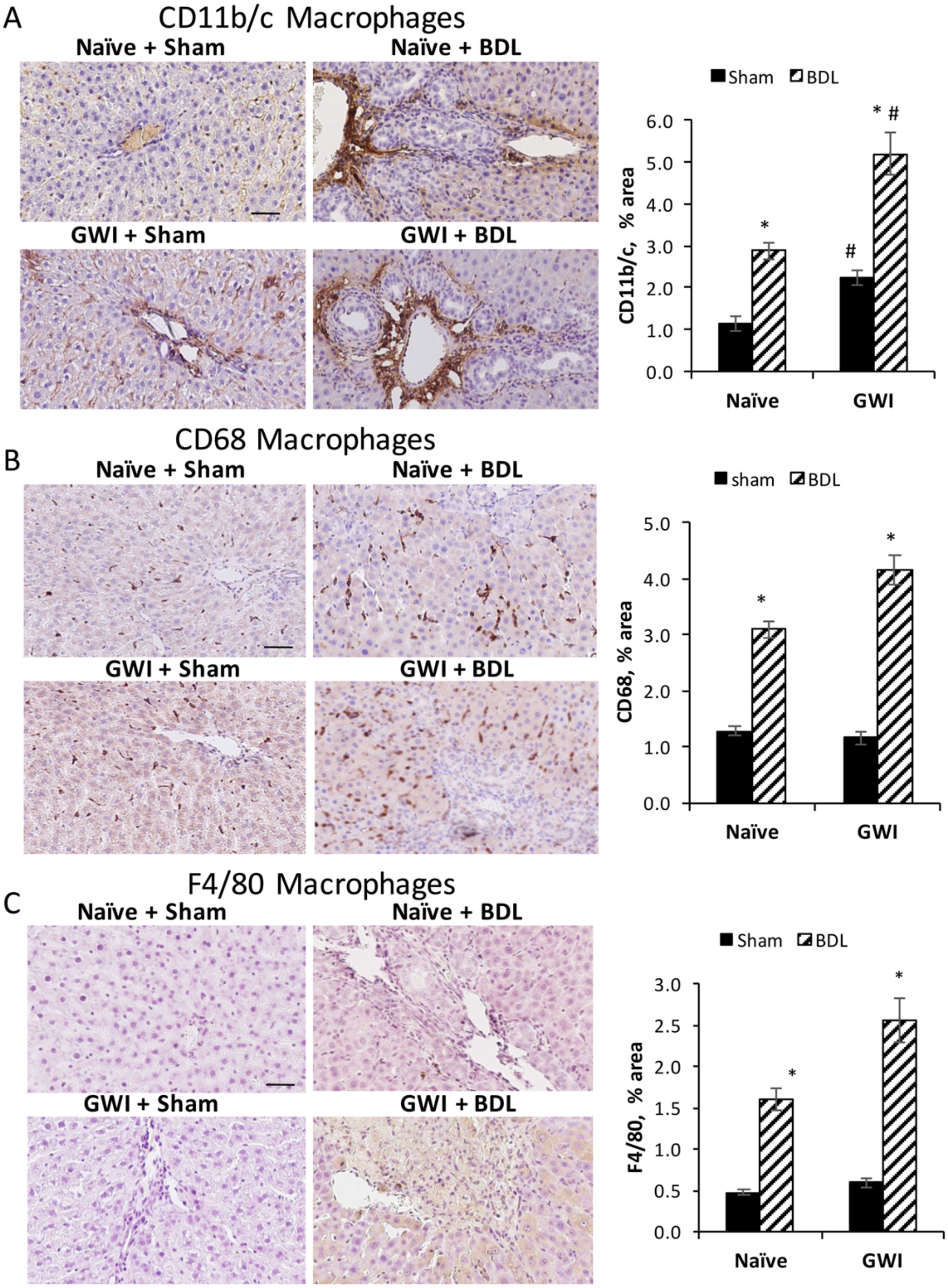

Supplementary Figure 1. IHC pictures and quantifications of CD11b/c⁺(panel A), CD68⁺ (panel B) and F4/80⁺ (panel C) macrophages in the livers of naïve and GWI rats subjected to sham or BDL surgery. $p<0.05$. * BDL vs Sham; #, GWI vs Naïve. Scale bar, 100μM.

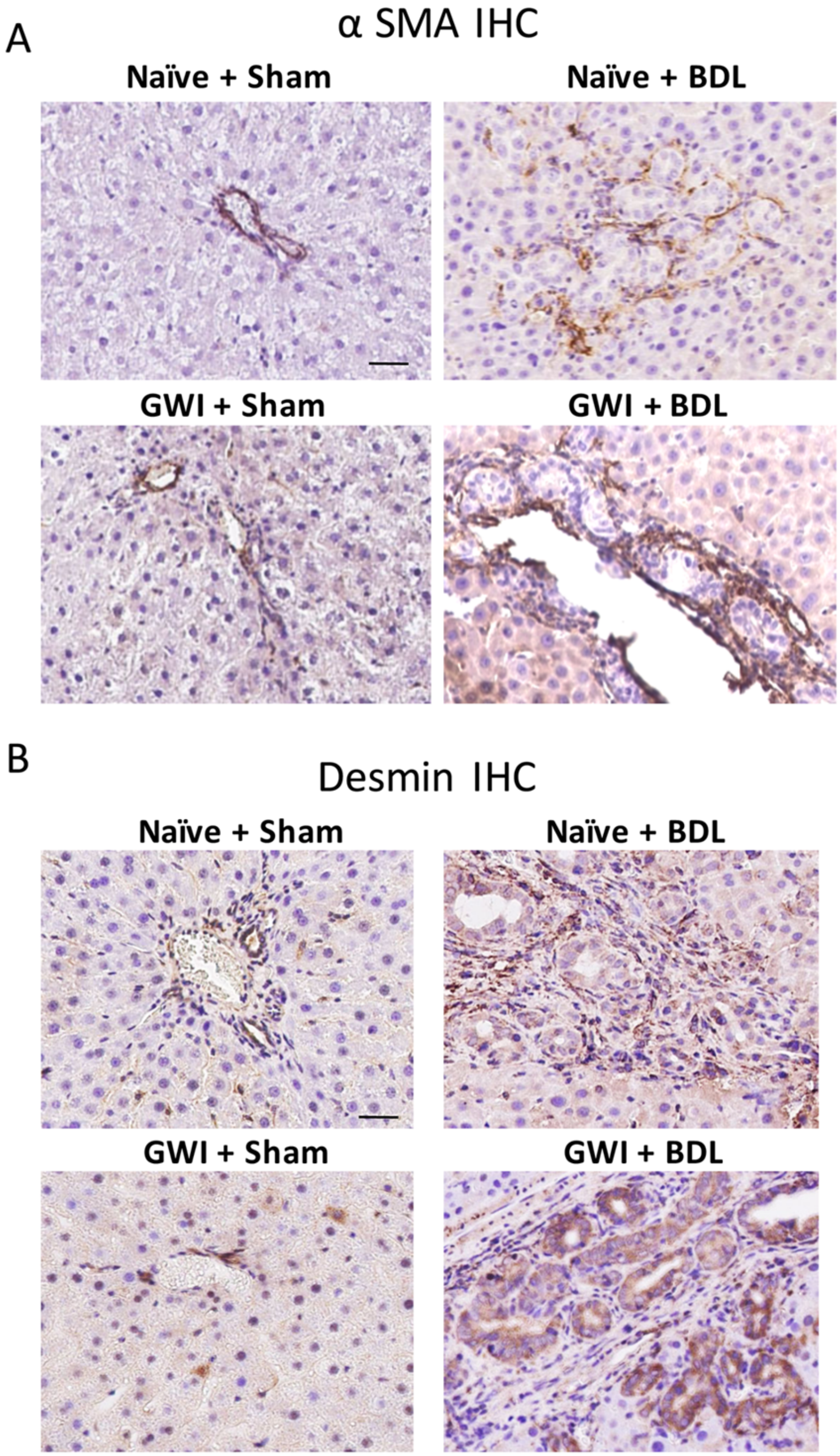

Supplementary Figure 2. Representative images of IHC of αSMA (panel A) and desmin (B) in the livers of naïve and GWI rats subjected to sham or BDL surgeries. Scale bar, 100μM.
